# Williams–Beuren Syndrome as a Potential Risk Factor for Burkitt Lymphoma

**DOI:** 10.3389/fgene.2018.00368

**Published:** 2018-09-04

**Authors:** Ryo Kimura, Yuko Ishii, Kiyotaka Tomiwa, Tomonari Awaya, Masatoshi Nakata, Takeo Kato, Shin Okazaki, Toshio Heike, Masatoshi Hagiwara

**Affiliations:** ^1^Department of Anatomy and Developmental Biology, Graduate School of Medicine, Kyoto University, Kyoto, Japan; ^2^Department of Pediatric Hematology/Oncology, Osaka City General Hospital, Osaka, Japan; ^3^Department of Child Neurology, Osaka City General Hospital, Osaka, Japan; ^4^Department of Pediatrics, Graduate School of Medicine, Kyoto University, Kyoto, Japan; ^5^Todaiji Ryoiku Hospital for Children, Nara, Japan

**Keywords:** williams–beuren syndrome, burkitt lymphoma, gene expression, non-hodgkin lymphoma, 7q11.23

## Abstract

Williams–Beuren syndrome (WBS) is a multisystemic neurodevelopmental disorder caused by a hemizygous deletion on chromosome 7q11.23. Though at present there is a limited number of reports on WBS patients with tumors, most cases are related to blood cancer in children with WBS. We describe a case of Burkitt lymphoma in a 21-year-old man with WBS. In addition to providing a summary of published reports describing tumors observed in patients with WBS, we present a hypothesis about a possible mechanism of oncogenesis. In particular, we identified some significantly dysregulated cancer-related genes using blood samples from this patient at the age of 19 years (who have not yet developed Burkitt lymphoma). Our findings may provide a new perspective on the relation between WBS and Burkitt lymphoma.

## Background

Williams–Beuren syndrome (WBS; MIM 194050) is a multisystemic neurodevelopmental disorder caused by a microdeletion of chromosome 7q11.23 (Pober, 2010). The prevalence of WBS is estimated as between 1 in 7500 and 1 in 20,000 individuals. WBS is characterized by a highly variable phenotype, including “elfin face" appearance, supravalvular aortic stenosis (SVAS), visuospatial deficits and hypersociability. Although there have been multiple case reports of pediatric patients with WBS developing non-Hodgkin lymphoma (NHL), as of yet there is no clear evidence of an association between WBS and lymphoma risk (Amenta et al., 2004; Thornburg et al., 2005; Onimoe et al., 2011; Zhukova and Naqvi, 2013; Guenat et al., 2014; Vanhapiha et al., 2014). It is possible that the low incidence of both these rare diseases conceals the relationship between them. NHL is a heterogeneous group of lymphoid malignancies, 85–90% of which arise from B lymphocytes (Shankland et al., 2012). Several different systems have been proposed for classifying NHL subtypes according to their histological characteristics. Burkitt lymphoma is a highly aggressive type of NHL and is the fastest growing human tumor (Molyneux et al., 2012). Burkitt lymphoma is classified into three subtypes: endemic, sporadic, and immunodeficiency-related Burkitt lymphoma. The sporadic type of Burkitt lymphoma occurs most commonly in children aged 3–12 years (median 6–8 years) in region such as North America, northern and eastern Europe, and east Asia and is 3.5 times more common in boys than in girls (Molyneux et al., 2012). However, the precise molecular mechanism underlying the relation between Burkitt lymphoma and WBS has been unclear. Here we provide a summary of published reports describing tumors in patients with WBS and report a case of Burkitt lymphoma in a young adult with WBS.

## Case Presentation

**FIGURE 1:**
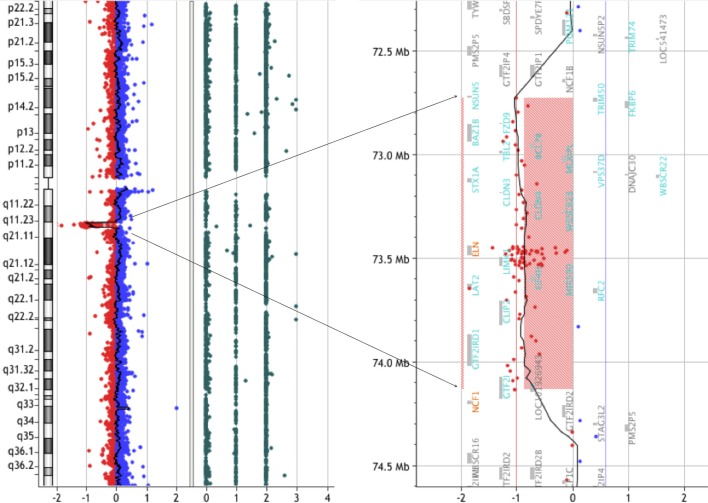
CGH array analysis of the microdeletion in chromosome seven of a WBS patient. CGH array profile of chromosome seven revealing the heterozygous deletion in 7q11.23.

We have been recruiting and following patients with WBS since 2012 in conjunction with our clinical research. In February 2014, a 19-year-old male was admitted to our hospital to take part in one of our clinical research projects. The patient had been clinically diagnosed with WBS at the age of 1 year based on typical clinical features, including the characteristic facial appearance. We performed array-CGH analysis and detected a deletion extending 1.4-Mb on chromosome 7q11.23 (**Figure 1**). Moreover, we did not find any pathological copy number variations (CNVs) other than 7q11.23. Laboratory data were normal: hemoglobin (Hb) 15.9 g/dL, MCV 83.6 fL, MCH 30.3 p., MCHC 36.3%, hematocrit (Hct) 43.8%, red blood cell (RBC) 5.24 × 10^6^/μL, white blood cell (WBC) 5.28 × 10^3^/μL, neutrophils 56%, lymphocytes 35%, eosinophils 0%, monocytes 9%, basophils 0%, and platelets 173 × 10^9^/L. The patient was followed as an outpatient in our hospital and was clinically assessed every 3 months. In October 2015, when the patient was 21 years of age, he was admitted to our hospital with acute abdominal pain. Abdominal computed tomography (CT) scans revealed ileocecal intussusception by mass. An ileocecal resection was performed. Immunohistochemistry (IHC) staining confirmed strong positivity of CD20, CD10, and c-MYC. Serologic tests for HIV and hepatitis B/C were all negative. Epstein-Barr-virus IgG and EBNA1 IgG was positive. EBER was negative. The biopsy confirmed the diagnosis of Burkitt lymphoma with a gene breakpoint on MYC (8q24) determined by FISH. Cytogenetic analysis revealed a karyotype of 46, XY, *t*(8;14) (q24.1;q32), add(13) (q22)[8]/46, XY [12]. The patient received conventional treatment for Burkitt lymphoma according to the LMB-96 group C protocol, plus rituximab. In order to avoid complications, the duration of therapy and doses of the agents were reduced. However, despite of intensive chemotherapy, the patient passed away in 2016.

## Materials and Methods

### Participants and Informed Consent

This study was conducted according to the Declaration of Helsinki; all participants provided written informed consent. The local ethics committees of Kyoto University Graduate School of Medicine and Osaka City General Hospital approved this study. The demographic characteristics of participants are summarized in **Supplementary Table S1**.

### DNA and RNA Isolation

Peripheral whole blood samples were taken and immediately stored in an EDTA collection tube and PAXgene Blood RNA tube (QIAGEN, Tokyo, Japan) for DNA and RNA extraction, respectively. Total DNA was isolated using a QIAamp DNA Blood Mini Kit (QIAGEN). RNA was extracted by using a PAXgene Blood RNA system (QIAGEN) following the manufacturer’s protocol. RNA quality was assessed using an Agilent 2100 Bioanalyzer (Agilent Technologies, Tokyo, Japan).

### CNV Analysis

Array-CGH was performed using an Agilent Sureprint G3 CGH+SNP 4 × 180 K microarray (G4890A; Agilent Technologies) following the manufacturer’s protocols. Briefly, 500 ng of purified DNA of a patient and a control (Agilent Technologies) were double-digested with RsaI and AluI enzymes (Agilent Technologies) for 2 h at 37°C. Each digested sample was labeled for 2 h with the Agilent Genomic DNA Labeling Kit, using Cy5-dUTP for the patient DNA and Cy3-dUTP for the control DNA. Labeled products were purified and prepared according to the Agilent protocol. After probe denaturation and pre-annealing with 50 ng of human Cot-1 DNA (Thermo Fisher Scientific, Yokohama, Japan), hybridization was performed at 65°C for 24 h in a rotating oven. After washing steps, the array slide was scanned with an Agilent Microarray Scanner (G2565CA). The spot intensities were measured and the image files quantified using the Agilent Feature Extraction 11.0.1.1 software. Text outputs from the quantitative analyses were imported into Agilent CytoGenomics 4.0 software (Agilent Technologies).

### Gene Expression Analysis

A High Capacity cDNA Reverse Transcription Kit (Thermo Fisher Scientific) was used to synthesize cDNA from 200 ng of total RNA. qPCR was performed on the cDNA using an ABI 7900 HT Fast Real-Time PCR System (Thermo Fisher Scientific). Raw threshold-cycle (Ct) values were obtained using SDS2.4 (Thermo Fisher Scientific). The relative quantification of gene transcripts was determined by the ddCt method. A standard curve was also generated to evaluate the efficiency of the qPCR experiment. Each sample was run in duplicate along with an endogenous control. We assessed the appropriate reference gene using TaqMan probes as endogenous controls. We selected GAPDH (Hs02758991_g1) as the reference gene for validation, including randomly selected genes. *P*-values were calculated by Student’s *t*-test. All primers used for the qRT-PCR analysis are listed in **Supplementary Table S2**.

## Discussion

**FIGURE 2:**
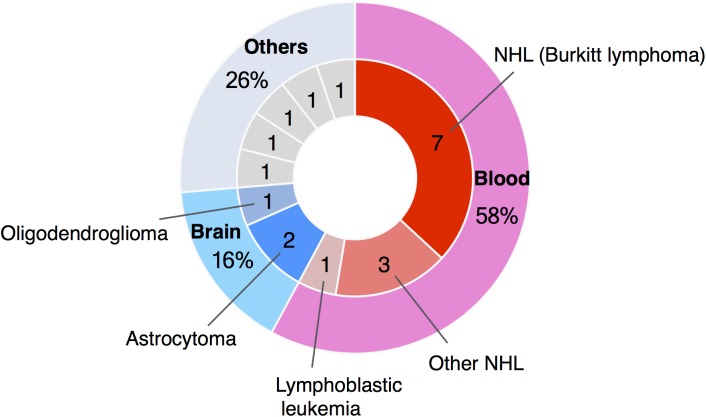
Summary of case reports of tumors in WBS patients. The number of reported cases of blood cancer in patients with WBS has reached 11, and seven of these were cases of Burkitt lymphoma.

Here we report a clinical profile and possible molecular mechanism of a 21-year-old male WBS patient who developed Burkitt lymphoma. Recently, the number of reports of blood cancer in patients with WBS has reached 11 cases, with 7 (64%) of these being Burkitt lymphoma (**Figure 2**). Burkitt lymphoma is the most common NHL of childhood (Hochberg et al., 2009). In agreement with these epidemiological data, Burkitt lymphoma in patients with WBS has been reported mostly in patients under 10 years (Amenta et al., 2004; Thornburg et al., 2005; Onimoe et al., 2011; Zhukova and Naqvi, 2013; Guenat et al., 2014; Vanhapiha et al., 2014). However, in adolescents, Burkitt lymphoma represents only approximately 20% of all NHL, and in young adults this figure drops to 5–10% (Hochberg et al., 2009).

Chromosomal translocation *t*(8;14) (q24; q32) activates the MYC oncogene and is the hallmark of Burkitt lymphoma (Molyneux et al., 2012). However, MYC deregulation alone seems to be insufficient for the malignant transformation, and additional synergistic mutations are required (Havelange et al., 2016). It is well known that GTF2I is located in the 7q11.23 region, which is interact with MYC (Roy et al., 1993). It is also known that Burkitt lymphoma cells express surface immunoglobulin M and B cell-associated antigens (CD19, CD20, CD22, and CD79a). CD19 expression in particular has been shown to promote MYC-dependent B cell lymphomagenesis (Rickert, 2013). B cell lymphomas frequently dysregulate B cell receptor (BCR) signaling, which plays an important role in B cell proliferation and survival. Burkitt lymphomas critically depend on the activation of “tonic” BCR signaling. BCR activation can be influenced by a specific immunoglobulin structure, the expression and mutations of adaptor molecules (such as BLNK), and the activity of kinases (such as SYK, PI3K) or phosphatases (such as PTEN) (Seda and Mraz, 2015). SWI/SNF or BAF complexes mediate ATP-dependent chromatin remodeling to regulate gene expressions that have recently been implicated in human malignancies (Kadoch et al., 2013; Kadoch and Crabtree, 2015). Alterations of SWI/SNF genes clearly play an important role in cancer development, progression, and/or resistance to therapy (Masliah-Planchon et al., 2015).

**FIGURE 3:**
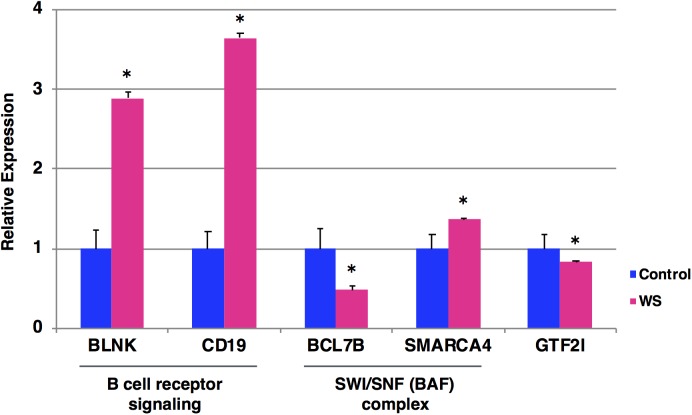
Expression levels of the selected five genes by qRT-PCR. The relative expression level of each gene was normalized to that of the internal control *GAPDH* from a WBS patient at the age of 19 years who have not yet developed Burkitt lymphoma (red) and five age/sex-matched control subjects (blue). Student’s *t*-test was used for the statistical analysis. ^∗^*P*-values < 0.05 were considered statistically significant.

To examine the expression levels of BCR signaling-related genes (*BLNK* and *CD19*), SWI/SNF genes (*BCL7B* and *SMARCA4*) and *GTF2I* gene, we performed qRT-PCR analysis of blood samples from a WBS patient at the age of 19 years (who have not yet developed Burkitt lymphoma) and five age/sex-matched control subjects. We found that BCR signaling-related genes (*BLNK* and *CD19*) were significantly upregulated in WBS compared to control subjects (**Figure 3**). In addition, SWI/SNF genes (*BCL7B* and *SMARCA*4) and *GTF2I* gene were significantly dysregulated in WBS relative to the control subjects. The *GTF2I* and *BCL7B* genes are located in the 7q11.23 region, which is commonly deleted in WBS. The *GTF2I* gene is known to be linked its deletion or missense mutation with cancerous phenotype (Petrini et al., 2014; Guenat et al., 2017). The human *BCL7* gene family consists of *BCL7A*, *BCL7B*, and *BCL7C*. A number of clinical studies have reported that members of the BCL7 family are involved in cancer incidence, progression, and development. A recent study suggested that BCL7B-deficiency confers a risk of several malignancies, such as WBS, through the Wnt Signaling pathway (Uehara et al., 2015; Decimi et al., 2016). On the other hand, changes in the expression levels of BCR signaling-related genes (*BLNK* and *CD19*) were more obvious than changes in *BCL7B* gene expression. These findings suggest that multiple WBS-related gene dysregulations may be involved in the increased incidence of lymphoma among individuals with WBS.

Burkitt lymphoma has a very high proliferation index, which makes prompt diagnosis and initiation of therapy very important to increase chances of survival (Shankland et al., 2012). Therefore, early detection and treatment are important issues in Burkitt lymphoma. Large and better-designed cohort studies are needed to confirm a possible relationship between WBS and Burkitt lymphoma.

## Concluding Remarks

To our knowledge, this is the first reported case of a young adult WBS patient with Burkitt lymphoma. Our findings will help to provide an insight into the relation between WBS and Burkitt lymphoma.

## Author Contributions

RK and MH analyzed and interpreted the patient data. YI, TK, SO, KT, and TH managed the patient regarding the hematological and neurodevelopmental aspects of the cases in this cohort. RK, MN, and TA performed experiments and analyzed the data. RK prepared the draft. All authors contributed to and approved the final manuscript.

## Conflict of Interest Statement

The authors declare that the research was conducted in the absence of any commercial or financial relationships that could be construed as a potential conflict of interest.
