# Supplementary material for: Williams–Beuren Syndrome as a Potential Risk Factor for Burkitt Lymphoma
Source: Front Genet. 2018 Sep 4;9:368. doi: 10.3389/fgene.2018.00368 (PMC6131482; doi:10.3389/fgene.2018.00368)
Supplement: Supplementary file 1 [file Table_1.DOCX]

Supplementary Material

Williams syndrome as a potential risk factor for Burkitt lymphoma

**Ryo Kimura^1^*, Yuko Ishii^2^, Kiyotaka Tomiwa^3,4,5^, Tomonari Awaya^1,4^, Masatoshi Nakata^1^, Takeo Kato^4^, Shin Okazaki^3^, Toshio Heike^4^, Masatoshi Hagiwara^1^**

*** Correspondence:** Ryo Kimura: kimura.ryo.2w@kyoto-u.ac.jp

# Supplementary Tables and Figures

**1. Supplementary Tables.**

**Table S1:** Demographic information of participants

| Group | Age (years) | Sex | Ethnicity |
| --- | --- | --- | --- |
| WS | 19 | M | Japanese |
| Control | 21 | M | Japanese |
| Control | 21 | M | Japanese |
| Control | 22 | M | Japanese |
| Control | 21 | M | Japanese |
| Control | 22 | M | Japanese |

**Table S2:** Taqman probe used in real-time RT-PCR analysis

| Gene Symbol | Assay ID | Gene Name |
| --- | --- | --- |
| *GAPDH* | Hs02758991_g1 | glyceraldehyde-3-phosphate dehydrogenase |
| *BLNK* | Hs00179459_m1 | B-cell linker |
| *CD19* | Hs01047410_g1 | CD19 molecule |
| *BCL7B* | Hs00892361_m1 | B-cell CLL/lymphoma 7B |
| *SMARCA4* | Hs00231324_m1 | SWI/SNF related, matrix associated, actin dependent regulator of chromatin, subfamily a, member 4 |
| *GTF2I* | Hs01073660_m1 | general transcription factor IIi |
